# Kisspeptin Is a Novel Regulator of Human Fetal Adrenocortical Development and Function: A Finding With Important Implications for the Human Fetoplacental Unit

**DOI:** 10.1210/jc.2017-00763

**Published:** 2017-06-21

**Authors:** Harshini Katugampola, Peter J. King, Sumana Chatterjee, Muriel Meso, Andrew J. Duncan, John C. Achermann, Leo Guasti, Lea Ghataore, Norman F. Taylor, Rebecca Allen, Shemoon Marlene, Joseph Aquilina, Ali Abbara, Channa N. Jaysena, Waljit S. Dhillo, Leo Dunkel, Ulla Sankilampi, Helen L. Storr

**Affiliations:** 1 Centre for Endocrinology, William Harvey Research Institute, London, EC1M 6BQ, United Kingdom; 2 Genetics & Genomic Medicine, UCL Great Ormond Street Institute of Child Health, London WC1N 1EH, United Kingdom; 3 Steroid Laboratory, Department of Clinical Biochemistry (Viapath Analytics), King’s College Hospital, London SE5 9RS, United Kingdom; 4 Fetal Medicine Centre, Royal London Hospital, Barts Health Trust, London E1 1BB, United Kingdom; 5 Section of Endocrinology and Investigative Medicine, Imperial College London, London W12 0NN, United Kingdom; 6 Department of Pediatrics, Kuopio University Hospital, Kuopio, Finland

## Abstract

**Context:**

The human fetal adrenal (HFA) is an integral component of the fetoplacental unit and important for the maintenance of pregnancy. Low kisspeptin levels during pregnancy are associated with miscarriage, and kisspeptin and its receptor are expressed in the HFA. However, the role of kisspeptin in fetal adrenal function remains unknown.

**Objective:**

To determine the role of kisspeptin in the developing HFA.

**Design:**

Experiments using H295R and primary HFA cells as *in vitro* models of the fetal adrenal. Association of plasma kisspeptin levels with HFA size in a longitudinal clinical study.

**Setting:**

Academic research center and tertiary fetal medicine unit.

**Participants:**

Thirty-three healthy pregnant women were recruited at their 12-week routine antenatal ultrasound scan.

**Main Outcome Measures:**

The spatiotemporal expression of *Kiss1R* in the HFA. The production of dehydroepiandrosterone sulfate (DHEAS) from HFA cells after kisspeptin treatment, alone or in combination with adrenocorticotropic hormone or corticotropin-releasing hormone. Fetal adrenal volume (FAV) and kisspeptin levels at four antenatal visits (∼20, 28, 34, and 38 weeks’ gestation).

**Results:**

Expression of *Kiss1R* was present in the HFA from 8 weeks after conception to term and was shown in the inner fetal zone. Kisspeptin significantly increased DHEAS production in H295R and second-trimester HFA cells. Serial measurements of kisspeptin confirmed a correlation with FAV growth in the second trimester, independent of sex or estimated fetal weight.

**Conclusions:**

Kisspeptin plays a key role in the regulation of the HFA and thus the fetoplacental unit, particularly in the second trimester of pregnancy.

## Teaser

Kisspeptin is a regulator of human fetal adrenocortical development and function. This finding suggests an important role for kisspeptin in the human feto-placental unit and maintenance of pregnancy.

Kisspeptins are a family of peptide hormones encoded by the *Kiss1* gene and are the endogenous ligands for the G-protein coupled receptor Kiss1R in humans (1, 2). Kiss1R is widely expressed in a number of human tissues, including the hypothalamus and pituitary (1–3). In the latter, kisspeptin–Kiss1R signaling has a vital role in the secretion of GnRH at puberty (4, 5). Kisspeptin and Kiss1R are robustly expressed in the syncytiotrophoblast cells of the placenta and may have an important role in the regulation of trophoblast invasion into the maternal uterine wall during placentation (1, 6, 7). In males and nonpregnant females, circulating kisspeptin levels are very low. Maternal circulating kisspeptin increases from around 8 weeks’ gestation∼940-fold and >7000 fold in the second and third trimesters, respectively (8). At ∼5 days postpartum, the levels fall to prepregnancy levels, implicating the placenta as the source of kisspeptin (8). Several studies suggest that low circulating maternal kisspeptin is associated with intrauterine growth restriction and preeclampsia (9, 10). Additionally, trophoblast expression of kisspeptin and its receptor in the first trimester is lower in women with recurrent miscarriage compared with normal pregnancies (11). Recently, a large prospective study demonstrated that plasma kisspeptin at the antenatal booking visit was lower in women who later had miscarriages than in those with normal pregnancies (12). Therefore, there is compelling evidence that decreased kisspeptin may be a biomarker of placental dysfunction in pregnancy and may also identify asymptomatic pregnant women at greater risk of miscarriage. However, the mechanisms underlying these associations are unknown.

The human fetal adrenal (HFA) cortex plays a critical role in the fetoplacental unit and a pivotal role in the endocrine control of pregnancy and parturition (13–15). The developing HFA consists of three zones: the centrally located fetal zone (FZ), the outer definitive zone (DZ), and the transitional zone (TZ) between the FZ and DZ (13, 16). At ∼8 to 10 weeks’ gestation [6 to 8 weeks post-conception (wpc)], there is rapid HFA growth caused by enlargement of the FZ, which accounts for most of the fetal adrenal mass by midgestation (16 to 20 weeks’ gestation, 14 to 18 wpc) (13, 16, 17). Except for transiently in the first trimester, FZ cells do not express the enzyme 3*β*HSD, needed for glucocorticoid and mineralocorticoid production. Therefore, HFA steroidogenesis is characterized by early transient cortisol production, which is then suppressed until late gestation (16, 17). The principal steroid output from the FZ in humans is dehydroepiandrosterone (DHEA), which is sulfated to DHEA sulfate (DHEAS) by the sulfotransferase family 2A member 1 gene *SULT2A1* before secretion (13). The placenta cannot produce estrogens *de novo* because it lacks the cytochrome P450 CYP17 enzyme (18). Therefore, the functional role of the HFA is to produce steroid precursors, which are converted to estrogens by the placenta (19). Placental estrogens are critical for intrauterine homeostasis, fetal maturation, and the activation of parturition (14, 15, 19). Consistent with this role, a disproportionate enlargement of the HFA gland FZ may accurately predict impending preterm birth (20, 21). Soon after birth, the HFA undergoes rapid involution, with rapid disappearance of the FZ and a decrease in androgen secretion (22, 23).

HFA development and function are complex and poorly understood. Although placental corticotropin-releasing hormone (CRH) and fetal pituitary adrenocorticotropic hormone (ACTH) play important roles, other locally produced or placenta-derived factors must also be involved (13). Interestingly, there is 50-fold higher expression of *Kiss1R* in the HFA compared with the adult adrenal, and *Kiss1R* expression has been confirmed in the DZ and TZ of the HFA (24). Additionally, kisspeptin stimulates aldosterone production in cultured human adrenocortical H295R cells (24). Therefore, the HFA may be an important target for the high levels of circulating kisspeptin in pregnancy.

We investigated the hypothesis that kisspeptin is a regulator of human adrenocortical development and function, acting directly on the HFA to regulate fetal adrenal steroidogenesis via its receptor, Kiss1R.

## Materials and Methods

### Ethical approval

These studies were approved by the Brighton and Sussex Research Ethics Committee (reference 12/LO/1755). For the Finnish tissue samples, ethical approval was obtained from the Ethics Committee of the Pohjois-Savo Health Care District, Finland, and a permit to study human autopsy tissues and resection material was obtained from the Finnish National Authority for Medicolegal Affairs.

### Adrenal tissues

Tissue blocks from human fetuses were obtained from therapeutic termination of pregnancy, miscarriage, stillbirth, or intrauterine death at autopsies performed at Kuopio University Hospital, Finland. Cryosections of frozen HFA tissue were obtained from the Medical Research Council–Wellcome Trust Human Developmental Biology Resource (HDBR) (London and Newcastle). Maternal informed consent and approval from the local National Health Authority Ethics Committees were obtained. The age of the fetal samples was estimated as previously described (25), and the embryos were staged according to the Carnegie staging classification system (26). All samples had a normal male or female karyotype (46,XY or 46,XX, respectively).

### Immunofluorescence studies

Fresh HFA tissue, collected in ice-cold phosphate buffer saline (PBS) (Sigma, Dorset, UK), was fixed in 4% paraformaldehyde, cryoprotected in 30% sucrose, and embedded in optimal cutting temperature compound (Fisher Scientific, Loughborough, UK). Sections (10 μm) were cut with a cryostat (Leica GM 1510S). Hematoxylin and eosin staining was performed according to standard procedures. For immunofluorescence studies, sections were blocked for 30 minutes with 10% normal goat serum in PBS and then incubated with primary antibody (or control solution) overnight at room temperature followed by 2 hours of incubation with donkey antirabbit secondary antibody (Jackson Immunoresearch, West Grove, PA). For negative controls, the primary antibody was omitted or preincubated with its peptide antigen (1 μg/mL) at room temperature with agitation for 30 minutes. Unbound antibody was removed by PBS–triton washes. Nuclei were stained with 4′,6-diamidino-2-phenylindole dihydrochloride, 1 μg/mL (Sigma Aldrich, Dorset, UK), and cover slips were secured with fluorescent mounting media (Dako, Cambridge, UK). Slides were visualized and images taken with the Zeiss LSM510 laser scanning confocal microscope and the ZEN 2011 lite edition software. For details of antibodies see Supplemental Table 1.

### Cell culture

Ian Mason of the University of Edinburgh donated the human adrenocortical carcinoma cell line (H295R; CRL-2128). Primary HFA cells were derived from tissue samples obtained from the HDBR, which were dissected and disaggregated by incubation at 37°C in serum-free DMEM/F12 Ham (1:1 ratio) media (DMEM/F12, Invitrogen, Paisley, UK) with 100 units/mL penicillin and 100 μg/mL streptomycin (Pen/Strep, ThermoFisher Scientific, Paisley, UK) and 2 mg/mL collagenase (Sigma Aldrich) for 90 minutes. Cells were then plated and grown as below. H295R and HFA cells were grown in DMEM/F12, with 2% Ultroser G (BioSepra, Borehamwood, UK), 1% ITS (1 mg/mL insulin, 0.55 mg/mL transferrin, and 0.5 µg/mL sodium selenite; Sigma Aldrich), and Pen/Strep (27).

### Complementary DNA synthesis and quantitative reverse transcriptase polymerase chain reaction

Total RNA was extracted from cultured H295R and HFA cells and human placental tissue with the RNeasy kit (Qiagen). HFA RNA and adult adrenal RNA were obtained from the HDBR tissues and a Human Total RNA Master Panel II (Clontech), respectively, and converted to complementary DNA (cDNA) via M-MLV reverse transcription according to the manufacturer’s instructions (Promega, Southampton, UK). Quantitative reverse transcriptase polymerase chain reaction (qPCR) was performed in triplicate (each sample) on a Stratagene Mx3000P thermocycler with KAPA SYBR fast qPCR master mix with 200 nM forward and reverse primers (sequences available on request). Data were analyzed in MxPro software (Stratagene, Stockport, UK). The threshold cycle value was quantified by interpolating the quantity from a standard curve made from a gel-purified amplicon. Data were normalized to *GAPDH* expression and presented as a proportional increase or decrease from the calibrator (placenta or adult adrenal normalized to a value of 1 for comparison).

### Cell treatments

H295R and HFA cells were seeded into six-well plates (Greiner Bio-One, Stonehouse, UK) and grown until 60% to 70% confluency, then incubated in 1 mL serum-free media overnight. One milliliter fresh serum-free media alone (untreated) or containing one of the following was added for 24 hours: 100 nM kisspeptin, 10 nM CRH, 10 nM ACTH, or 10 µM forskolin (Sigma Aldrich). CRH and ACTH directly stimulate DHEAS production in HFA cells (15, 28). Forskolin treatment directs steroid production toward the androgen pathway in the H295R adrenocortical tumor cell line (27). DHEAS was measured in the cell media by enzyme-linked immunosorbent assay (ELISA) (Demeditec Diagnostics, Kiel, UK), and the assay quality was checked for some experiments by liquid chromatography–tandem mass spectrometry (LC-MS/MS). LC-MS/MS is the gold standard method for quantifying steroid production; however, mass spectrometry results were unavailable for all the time points of interest. The kisspeptin concentration used was supraphysiological (100× maternal circulating concentrations). This concentration was chosen on the basis of previously published data (24) and dose response studies that showed a significant increase in DHEAS compared with untreated H295R cells with 10 nM (*P* < 0.01) and 100 nM (*P* < 0.05). Only 100 nM kisspeptin produced a significant increase in DHEAS compared with no treatment in 8- to 10-wpc (*P* < 0.05) and 15- to 20-wpc (*P* < 0.0001) HFA cells [Supplemental Figs. 1(A), 1(B), and 1(C), respectively].

### Protein assay

The cell lysate protein concentrations were determined by Bradford assay (29). Absorbance of each well at 595 nm (OD595) was determined with the PerkinElmer Wallac Victor2 1420 microplate reader. The protein content of each sample was determined as previously described (29).

### Immunoblotting

Kiss1R expression was assessed by immunoblotting after treatment of cells with kisspeptin. Cells were lysed in 200 μL radioimmunoprecipitation assay buffer (Sigma-Aldrich) containing protease inhibitor (cOmplete, Mini, EDTA-free Protease Inhibitor Cocktail Tablets, Roche, Welwyn Garden City, UK). Samples were heated to 95°C for 5 minutes in Laemmli buffer (Sigma-Aldrich) and loaded on 4% to 12% SDS-PAGE gels (Invitrogen). Size-separated proteins were then transferred to a nitrocellulose membrane, and the blots were immunolabeled overnight with the anti-Kiss1R antibody at 1:500 dilution or mouse monoclonal *β*-actin at 1:10,000 dilution (Sigma) as a loading control. Visualization of the proteins was performed with Alexa Fluor 680 and 800 secondary antibodies (Invitrogen) at a 1:5000 dilution and the Li-CoR Odyssey system.

### DHEAS quantification

Cell media were collected from treated cells, and DHEAS measurements were obtained with either the DHEAS ELISA Kit (Demeditec Diagnostics) or LC-MS/MS according to an optimized protocol (Supplemental Methods and Supplemental Tables 2–4). Experiments were performed in triplicate and repeated at least three times. The DHEAS results were corrected to the protein content of the attached cells, quantified by Bradford assay.

### Clinical study design and recruitment

A prospective observational study of patients with singleton, uncomplicated pregnancies was undertaken. Women attending their routine antenatal ultrasound scan (USS) at ∼12 weeks’ gestation at the Royal London Hospital, London between February 2013 and April 2014 were recruited. Informed consent was obtained, and translators were provided as necessary. Gestational age (GA) was established during the USS evaluation. Exclusion criteria included multiple pregnancy, coexistent maternal medical conditions (hypertension, preeclampsia, diabetes, thyroid, adrenal or renal disease), congenital fetal abnormalities or chromosomal anomalies, maternal infection (including HIV), maternal alcohol abuse, heavy smoking (>10 cigarettes daily before pregnancy or >5 cigarettes daily during pregnancy), and maternal exposure to psychotropic medications. Serial measurements of fetal adrenal size were performed at the time of the routine anomaly scan (∼20 weeks’ gestation, visit 1) and at 3 other time points: ∼28, 34, and 38 weeks’ gestation (visits 1, 2, and 3, respectively) (Table 1). Maternal plasma samples for kisspeptin were taken at the same time points as the USS, and subjects were followed up until the outcome of pregnancy was known.

**Table 1.** **Patient Characteristics, Timing of Antenatal Assessments, and Pregnancy Outcomes**

Patient Characteristic	Mean ± SD	Range	N
Age, y	26.8 ± 6.1	17.0 to 37.0	33
Body mass index, kg/m^2^	25.4 ± 5.3	18.8 to 41.1	33
GA, wk			
Visit 1	20.35 ± 0.68	19.29 to 22.57	31
Visit 2	28.07 ± 0.75	26.29 to 29.86	32
Visit 3	34.42 ± 0.75	33.00 to 36.14	28
Visit 4	38.09 ± 0.62	36.00 to 39.71	22
Gestation at delivery, wk	39.33 ± 2.83	26.86 to 42.14	31
BW, g	3006 ± 650	950 to 3680	31
BW, SDS	−0.698 ± 0.913	−3.30 to 0.840	31

N = total number of subjects assessed.

Abbreviation: SDS, standard deviation score.

### Subject details and pregnancy outcome

Thirty-three pregnant women of mean age 26.8 ± 6.1 years were recruited. The median gravidity and parity of the women were 1 (range 1 to 4) and 0 (range 0 to 2), respectively. The patient demographics are detailed in Table 1 and Supplemental Table 5. Two subjects (21, 23) moved from the area before completion of the study (after visits 2 and 3, respectively). Two babies were born prematurely at 26 and 33 weeks’ gestation (subjects 18 and 30, respectively) with normal birth weights (BWs) of 950 and 1340 g [BW standard deviation score (SDS) 0.84 and −1.95], respectively. Two infants (6, 22) born at 39.3 and 37.1 weeks’ gestation were small for GA, BW 2420 and 1720 g (BW SDS −2.42 and −3.30), respectively. The remaining subjects had term deliveries, mean gestation 39.96 ± 1.18 weeks (range 37.14 to 42.14 weeks) and BW SDS −0.71 ± 0.87 (−3.30 to 0.57).

### Fetal adrenal volume calculation

Fetal USS were performed by two fetal medicine doctors (R.A., S.M.) independently using the Voluson 730 and E8 systems (Voluson Expert, Milwaukee, WI). Two-dimensional measurements were taken in the transverse, coronal, and sagittal planes to obtain the length, width, and depth of the total adrenal gland and the FZ. Three-dimensional ultrasonography was performed to obtain the fetal adrenal volume (FAV), which was calculated with Virtual Organ Computer-Aided Analysis, 4D view software (General Electrical Medical Systems) (30). FAV data were missing for several patients who did not attend USS appointments (Supplemental Table 5).

### Measurement of plasma kisspeptin

Samples were stored at −20°C for between 6 and 18 months before kisspeptin measurements. Plasma kisspeptin immunoreactivity was measured at Imperial College, London with the established in-house assay (31, 32).

### Data analysis and statistics

*In vitro* data were evaluated via a paired two-tailed Student *t* test or one-way analysis of variance followed by a *post hoc* Tukey comparisons test (GraphPad Prism 6, San Diego, CA). All experiments were performed in triplicate and represent three or four independent experiments; error bars depict the standard deviations (SDs) of each individual experiment. Nonparametric continuous variables (FAV and kisspeptin levels) were analyzed via a Kruskal–Wallis test with Dunn–Bonferroni *post hoc* multiple comparison test correction. Continuous parametric variables (FAV in male and female fetuses) were compared via Student *t* test. *R* values are Pearson correlation coefficient (SPSS version 23; IBM Corp., Armonk, New York). *P* values <0.05 were statistically significant.

## Results

### Expression of Kiss1R in the developing HFA cortex

**Figure 1.**
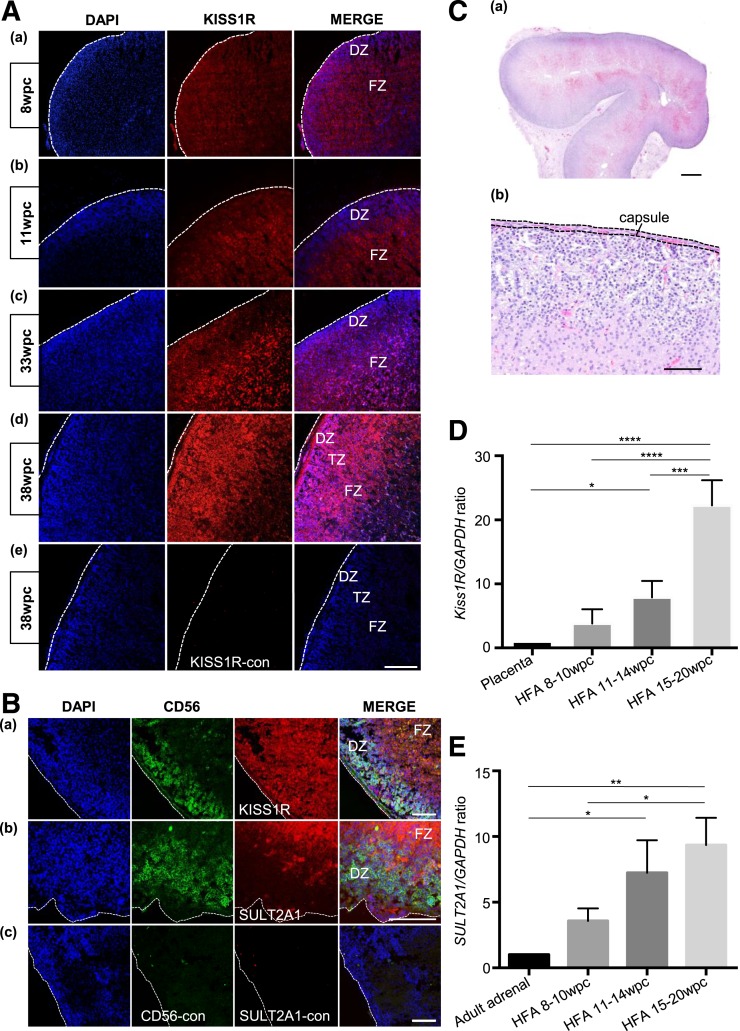
Expression of Kiss1R in the developing HFA cortex. (A) Immunofluorescence studies of the HFA from 8 wpc to term (panel a, 8 wpc; b, 11 wpc; c, 33 wpc; d and e, 38 wpc). Localization of Kiss1R (red) in the DZ, TZ, and throughout the FZ (panels a–d). No immunoreactivity is detected in the negative control with antigen (Kiss1R) preincubation (Kiss1R-con, panel e), demonstrating specificity of the Kiss1R antibody. The adrenal cortex is surrounded by an outer mesenchymal capsule (dashed line). Scale bar: 100 μm. (B) Colocalization studies of HFA at 12 wpc (panels a–c). CD56 (green) is expressed in the outer DZ/TZ (panels a and b) and SULT2A1 (red) in the inner FZ (panel b). Kiss1R (red) is seen throughout the cortex (FZ and DZ) (panel a). No immunoreactivity detected in the negative controls where the primary antibodies (CD56 and SULT2A1) were omitted (panel c, CD56-con, SULT2A1-con). The capsule is shown (dashed line). Scale bar: 100 μm. (C) Hematoxylin and eosin staining of HFA 33 wpc. A thin capsule (c) surrounds the outer DZ. The DZ of the cortex is the most superficial layer with closely packed, darker-stained cells. The deeper layer with the more eosinophilic appearance is the FZ. The TZ lies between the outer DZ and inner FZ. Scale bar: 1 mm (panel a), 100 μm (panel b). (D) *Kiss1R* qPCR was performed on cDNA obtained from three HFA samples from each stage of development (8 to 10 wpc, 11 to 14 wpc, 15 to 20 wpc). Placental cDNA was used as a positive control. Data points represent the mean ± SD from four independent experiments performed in triplicate. (E) *SULT2A1* qPCR was performed on cDNA obtained from three HFA samples from each stage of development (8 to 10 wpc, 11 to 14 wpc, 15 to 20 wpc). Adult adrenal cDNA was used as a positive control. Data points represent the mean ± SD from three independent experiments performed in triplicate. (D, E) Data are normalized to *GAPDH* expression and presented as a proportional increase or decrease from the calibrator (placenta and adult adrenal, normalized to a value of 1 for comparison). *P < 0.05; **P < 0.01; ***P < 0.001; ****P < 0.0001.

Immunohistochemical analysis demonstrated high expression of Kiss1R throughout the HFA cortex in all the GAs examined (8 wpc to term) [Fig. 1(A), panels a–d]. Colocalization studies using CD56 and SULT2A1 as markers of the DZ/TZ and FZ, respectively, verify Kiss1R expression in all three zones of the HFA [Fig. 1(B)]. Steroidogenic cells are identified by SF1 immunoreactivity throughout the HFA, and there is colocalization of SF1 and Kiss1R [Supplemental Fig. 1(D)]. Densely packed nuclei below the capsule and the larger cells in the center of the adrenal cortex have the appearance of DZ and FZ cells, respectively, and the TZ lies between these two zones [Fig. 1(C)].

### Quantitative assessment of Kiss1R expression in the developing HFA

*Kiss1R* and *SULT2A1* are highly expressed in the human placenta and adrenal gland, respectively. qPCR performed on first- and second-trimester HFA cDNA (8 to 10 wpc, 11 to 14 wpc, 15 to 20 wpc) showed an increase in both *Kiss1R* [Fig. 1(D)] and *SULT2A1* [Fig. 1(E)] messenger RNA (mRNA) expression with increasing GA. *Kiss1R* mRNA was significantly higher in 11- to 14-wpc HFA (7.9-fold, *P* < 0.05) and 15- to 20-wpc HFA (22.3-fold, *P* < 0.0001) than placenta [Fig. 1(D)]. At 8 to 10 wpc *Kiss1R* mRNA was 3.7 times higher in HFA than in human placenta, although this difference was not significant [Fig. 1(D)]. There was also a significant increase in *Kiss1R* expression from 8 to 10 wpc to 15 to 20 wpc (5.9-fold, *P* < 0.0001) and from 11 to 14 wpc to 15 to 20 wpc (2.8-fold, *P* < 0.001). The twofold increase in *Kiss1R* expression between 8 to 10 wpc and 11 to 14 wpc was not significant.

*SULT2A1* mRNA was significantly higher in 11- to 14-wpc (7.3-fold, *P* < 0.05) and 15- to 20-wpc HFA (9.4-fold, *P* < 0.01) than in human adult adrenal [Fig. 1(E)]. At 15 to 20 wpc, *SULT2A1* mRNA was 2.6 times higher in HFA than at 8 to 10 wpc (*P* < 0.05). At 8 to 10 wpc, *SULT2A1* mRNA was 3.6 times higher in HFA than in adult adrenal, although this result was not significant [Fig. 1(E)]. The 2.0- and 1.3-fold increases in *SULT2A1* expression from 8 to 10 wpc to 11 to 14 wpc and from 11 to 14 wpc to 15 to 20 wpc, respectively, were also not significant.

### The effect of kisspeptin treatment on *Kiss1R* mRNA and protein expression in H295R and HFA cells

**Figure 2.**
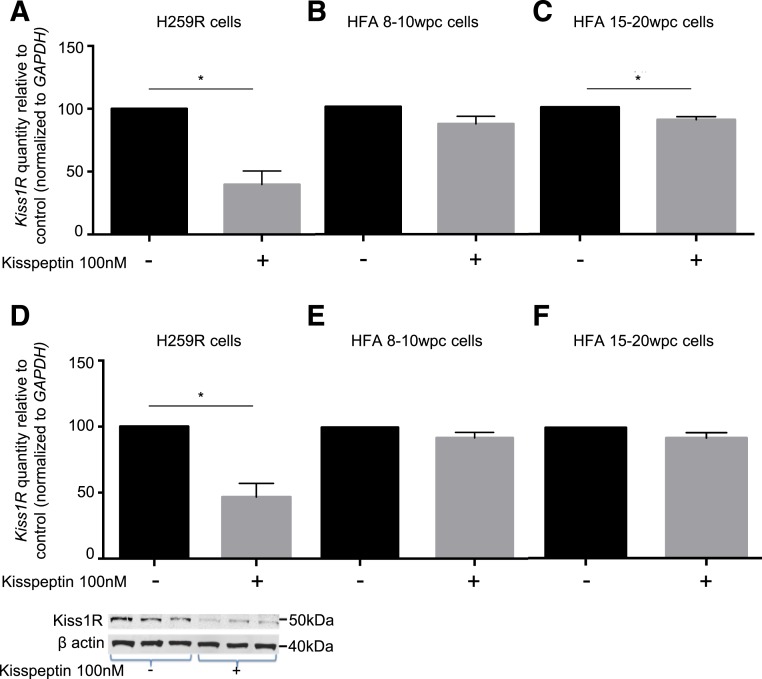
The effect of kisspeptin treatment on *Kiss1R* mRNA and protein expression in H295R and primary fetal adrenocortical cells. (A) Treatment with 100 nM kisspeptin for 24 hours significantly reduced *Kiss1R* mRNA expression in H295R cells. This effect was also evident in (C) 15 to 20 wpc HFA cells but not (B) 8 to 10 wpc HFA cells. Data are normalized to *GAPDH* expression and presented as a proportional increase or decrease from the control (unstimulated cells, normalized to a value of 100 for comparison). Data points represent the mean ± SD from three independent experiments performed in triplicate. (D–F) Densitometric analysis of Western blots showed a significant reduction in Kiss1R protein after treatment of (D) H295R cells with 100 nM kisspeptin for 24 hours but not (E) 8- to 10-wpc HFA cells or (F) 15- to 20-wpc HFA cells. Data points represent the mean ± SD from three independent experiments performed in triplicate. *β*-actin was used as a loading control. *P < 0.05.

Treatment of H295R adrenocortical cells with kisspeptin resulted in a significant (60%) decrease in *Kiss1R* mRNA expression [Fig. 2(A); *P* < 0.05]. A significant decrease (34%) in *Kiss1R* mRNA expression was also observed in 15- to 20-wpc HFA cells [Fig. 2(C); *P* < 0.05] but not 8- to 10-wpc HFA cells [16% decrease, Fig. 2(B)] in response to kisspeptin treatment. Kisspeptin treatment also resulted in a significant decrease in Kiss1R protein levels in H295R adrenocortical cells (53.5% decrease from baseline; *P* < 0.05) [Fig. 2(D)]. Kiss1R protein expression was decreased in 8- to 10-wpc HFA cells [8.3% reduction; Fig. 2(E)] and 15- to 20-wpc HFA cells [8.9% reduction; Fig. 2(F)] in response to kisspeptin treatment; however, these differences were not significant.

### The effect of kisspeptin and known adrenal regulators on DHEAS production (ELISA) in H295R and HFA cells

**Figure 3.**
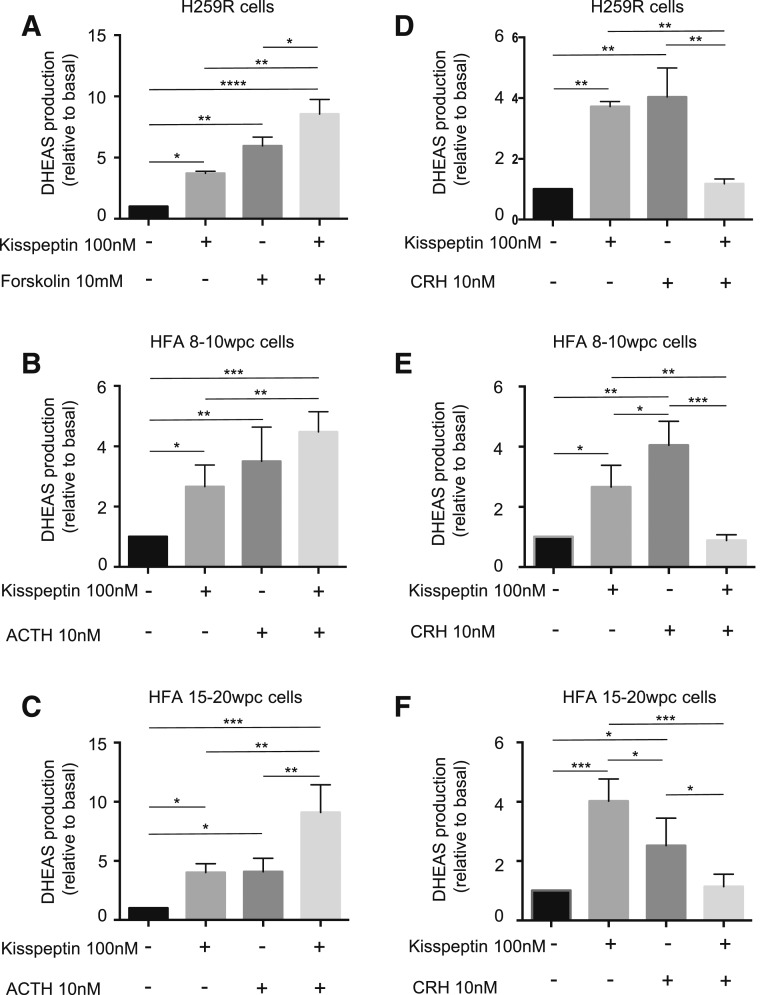
The effect of kisspeptin and known adrenal regulators on DHEAS production (ELISA) in H295R and primary fetal adrenocortical cells. (A–F) DHEAS production (ELISA) by H295R and HFA cells after kisspeptin and CRH treatments. (A) H295R cells were incubated for 24 hours with 100 nM kisspeptin and 10 μM forskolin individually or together; (B) 8- to 10-wpc HFA cells and (C) 15- to 20-wpc HFA cells were incubated for 24 hours with 100 nM kisspeptin and 10 nM ACTH individually or together. (D–F) DHEAS production (ELISA) by H295R and HFA cells after kisspeptin and CRH treatments. (D) H295R cells, (E) 8- to 10-wpc HFA cells, and (F) HFA cells 15 to 20 wpc were incubated for 24 hours with 100 nM kisspeptin or 10 nM CRH individually or together. Data points are mean ± SD from three independent experiments run in triplicate and expressed as the fold over basal level (normalized to a value of 1). −, no treatment; +, treatment added. *P < 0.05; **P < 0.01; ***P < 0.001; ****P < 0.0001.

Kisspeptin significantly increased DHEAS secretion from H295R, 8- to 10-wpc and 15- to 20-wpc HFA cells 3.7-fold [*P* < 0.05; Fig. 3(A)], 2.5-fold [*P* < 0.05; Fig. 3(B)], and 4.0-fold [*P* < 0.05; Fig. 3(C)] compared with untreated cells, respectively. DHEAS production from 8- to 10-wpc and 15- to 20-wpc HFA cells after kisspeptin treatment was similar to that produced by ACTH [3.5-fold (*P* < 0.01) and 4.1-fold (*P* < 0.05) compared with untreated cells, respectively]. Kisspeptin with forskolin increased DHEAS secretion 8.6-fold from H295R cells compared with untreated cells (*P* < 0.0001), which was 2.3 times (*P* < 0.01) and 1.4 times (*P* < 0.05) higher than kisspeptin or forskolin alone, respectively [Fig. 3(A)]. Kisspeptin with ACTH also increased DHEAS production 4.5-fold [*P* < 0.001; Fig. 3(B)] and 9.1-fold [*P* < 0.0001; Fig. 3(C)] from 8- to 10-wpc and 15- to 20-wpc HFA cells compared with untreated cells, respectively. This was 1.7 times [*P* < 0.05; Fig. 3(B)] and 2.3 times [*P* < 0.01; Fig. 3(C)] higher than with kisspeptin alone in 8- to 10-wpc HFA cells and 15- to 20-wpc HFA cells, respectively and 2.2 times [*P* < 0.01; Fig. 3(C)] higher than with ACTH alone in 15- to 20-wpc HFA cells.

DHEAS production by H295R, 8- to 10-wpc and 15- to 20-wpc HFA cells after CRH treatment was similar to that produced by ACTH and kisspeptin treatment alone (4.0-fold; *P* < 0.01; 4.0-fold, *P* < 0.001; and 2.5-fold, *P* < 0.05, compared with untreated cells, respectively) [Figs. 3(D–F)]. Compared with kisspeptin and CRH alone, treatment of H295R cells with a combination of kisspeptin and CRH resulted in a significant decrease (2.6-fold; *P* < 0.01 and 2.8-fold; *P* < 0.01) in DHEAS production, respectively [Fig. 3(D)]. The same pattern was observed in 8- to 10-wpc HFA cells (3.1-fold, *P* < 0.01; and 4.7-fold, *P* < 0.001, decrease of DHEAS compared with kisspeptin and CRH treatment alone, respectively) [Fig. 3(E)] and 15- to 20-wpc HFA cells (3.5-fold, *P* < 0.001, and 2.2-fold, *P* < 0.05, decrease of DHEAS compared with kisspeptin and CRH treatment alone, respectively) [Fig. 3(F)].

### The effect of kisspeptin on DHEAS production (LC-MS/MS) in H295R and HFA cells

DHEAS levels measured by LC-MS/MS increased 8.3-fold (H295R; *P* < 0.05) and 93.2-fold (8- to 10-wpc HFA cells; *P* < 0.05) after kisspeptin treatment [Supplemental Figs. 1(E) and 1(F) and Supplemental Tables 4(A) and 4(B)]. These DHEAS increases are 2.2 and 34.5 times higher compared with the ELISA, respectively. The lower calculated change observed by the ELISA compared with the LC-MS/MS assay in 8- to 10-wpc HFA cells can be attributed to the higher baseline values. This is likely to be caused interference in the ELISA at baseline from other steroid sulfates generated by the HFA tissue.

### FAV and kisspeptin levels in uncomplicated singleton pregnancies

**Figure 4.**
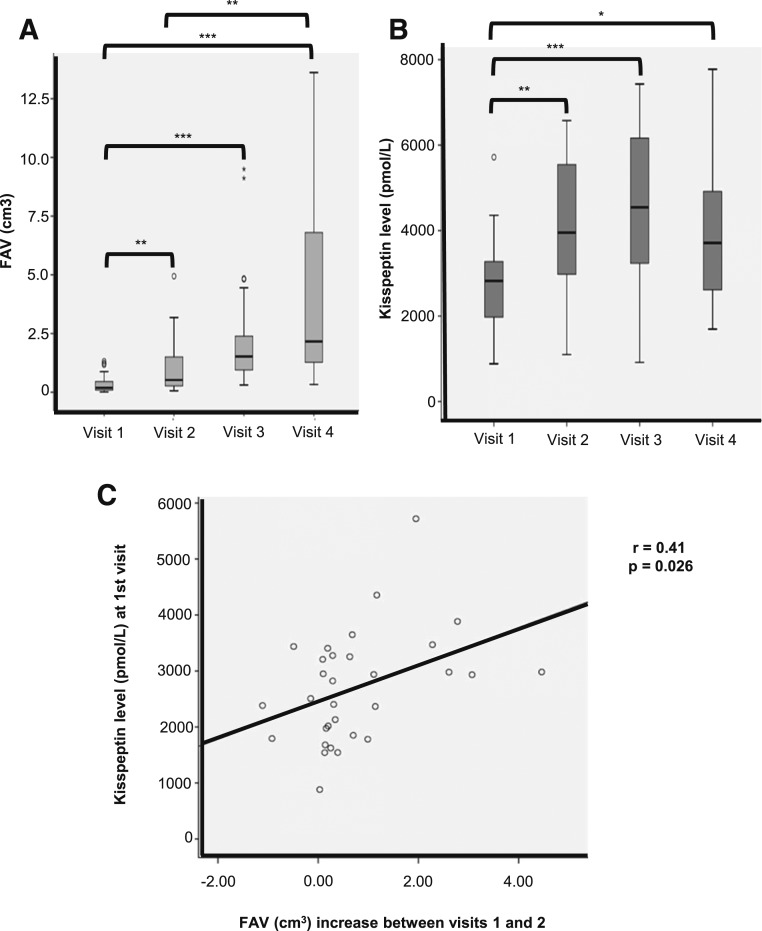
FAVs and kisspeptin levels in the maternal circulation during pregnancy. (A, B) Box and whisker plots of (A) FAVs (in cm^3^) and (B) maternal serum kisspeptin levels at the four antenatal visits (visit 1, 19 to 20 weeks; visit 2, 26 to 28 weeks; visit 3, 34 to 35 weeks; visit 4, 37 to 40 weeks). Box plots show the median, upper, and lower quartiles and IQRs. Open circles, outliers; star, extreme outliers. *P < 0.05; **P < 0.01; ***P < 0.001. (C) Scatterplot showing correlation between the kisspeptin level at the first visit and the increase in FAV between the first and second antenatal visits. *r*, Pearson coefficient.

FAVs increase steadily during pregnancy [Fig. 4(A) and Supplemental Table 5]. Median FAVs were 0.19 cm^3^ (interquartile range 0.08 to 0.48; n = 31), 0.52 cm^3^ (0.26 to 1.53; n = 32), 1.52 cm^3^ (0.94 to 2.40; n = 28), and 2.16 cm^3^ (1.17 to 7.87; n = 23) at antenatal visits 1 through 4, respectively. The range of FAVs increases as gestation advances, but there are significant increases in FAV between visits 1 and 2 (*P* < 0.01), visits 1 and 3 (*P* < 0.001), visits 1 and 4 (*P* < 0.001), and visits 2 and 4 (*P* < 0.01). Median kisspeptin levels were 2822 pmol/L (interquartile range 1913 to 0.48; n = 33), 3953 pmol/L (2823 to 5615; n = 31), 4545 pmol/L (3182 to 6182; n = 30), and 3711 pmol/L (2546 to 4937; n = 26) at antenatal visits 1 through 4, respectively [Fig. 4(B)]. There is considerable overlap of kisspeptin levels as gestation advances, but significant increases are noted between visits 1 and 2 (*P* < 0.05), visits 1 and 3 (*P* < 0.001), visits 1 and 4 (*P* < 0.001), and visits 2 and 4 (*P* < 0.01) [Fig. 4(B)].

### Relationship between FAV and plasma kisspeptin in singleton pregnancies

To corroborate the *in vitro* data, we assessed the association of the maternal kisspeptin levels with the subsequent FAV increment at the four different time points. The mean increase in FAV between antenatal visits 1 and 2 correlated with the kisspeptin level at visit 1 (*r* = 0.41, *P* = 0.026) [Fig. 4(C)], suggesting that the kisspeptin levels between 19 and 20 weeks’ gestation (17 to 18 wpc) may influence FAV increase between 19 and 28 weeks’ gestation (17 to 26 wpc). There was no significant difference in the mean rise of FAV between the first and second antenatal visits in male 0.83 ± 0.94 (n = 18) and female 1.28 ± 1.38 (n = 12) infants, respectively (*P* = 0.30; 95% CI, −0.42 to 1.31). There was no significant correlation between FAV and estimated fetal weight (efw) between the first and second antenatal visits (*r* −0.166; *P* = 0.36). Therefore, the significant correlations between maternal kisspeptin and FAV were independent of fetal sex and efw, suggesting that kisspeptin may be important for FA development in midpregnancy. There was no significant correlation between the maternal kisspeptin levels and the subsequent FAV increment at the other antenatal time points.

## Discussion

Circulating kisspeptin levels increase dramatically during pregnancy (8) and may play an important role in placentation by regulating placental invasion into the maternal uterine wall (1, 3, 6). Circulating kisspeptin levels are reduced in women with intrauterine growth retardation and preeclampsia (9, 33), and low maternal levels in early pregnancy have been associated with greater miscarriage risk (12). Therefore, kisspeptin may be an endocrine marker of functional placental tissue, and low placental kisspeptin may be associated with serious obstetric complications. The role of kisspeptin in pregnancy and the mechanisms underlying these associations are unclear.

Kisspeptin and its receptor, Kiss1R, are expressed in the central nervous system, pancreas, adipose tissue, testes, and spleen (2, 3). One other group has assessed the expression and localization of Kiss1R in the HFA (24). This study showed robust expression of *Kiss1R* mRNA in HFA tissue and Kiss1R protein expression in the DZ and TZ of 14 to 36 weeks’ gestation HFA tissues by immunohistochemistry. We confirmed Kiss1R protein expression in the HFA cortex from 8 wpc (10 weeks’ gestation) to term by immunofluorescence. Interestingly, it was identified throughout the adrenal cortex, with expression in the inner FZ as well as the outer DZ and TZ. The reason for this discrepancy is unclear but may be accounted for by the different methods and antibodies used. Additionally, our immunofluorescence and *in vitro* data concord as presumably, kisspeptin stimulates DHEAS production from FZ cells. Consistent with this finding, SULT2A1 (DHEA sulfotransferase) is localized to the FZ and converts DHEA to DHEAS. This finding suggests that the FZ may be an important target for kisspeptin during pregnancy.

Quantitative evaluation shows that *Kiss1R* mRNA expression increases significantly in midgestation (11 to 20 wpc); therefore, this may be a critical time point for the action of kisspeptin on the FZ. The production of DHEAS begins at ∼8 to 10 weeks’ gestation (6 to 8 wpc) but increases in the second and third trimesters (13). The increase in *Kiss1R* expression in midgestation (11 to 20 wpc) is paralleled by an increase in *SULT2A1* mRNA expression. Taken together, these data suggest that the FZ is an important target for kisspeptin, particularly in the second trimester.

Kiss1R is a G-protein coupled receptor. Kisspeptin–KISS1R signaling is best characterized in GnRH neurons, and in these cells KISS1R undergoes both kisspeptin-triggered and kisspeptin-independent signaling, internalization, and recycling (34). This process ensures a dynamic population of functional cell surface receptors and tight regulation of the biochemical response. In HFA cells, kisspeptin treatment resulted in a significant decrease in *Kiss1R* mRNA expression in adrenocortical tumor (H295R) and second-trimester HFA cells. This decrease was paralleled by a significant decrease in Kiss1R protein levels in H295R cells. We hypothesize that high circulating kisspeptin levels may downregulate *Kiss1R* expression in the HFA to regulate signaling and therefore ensure tight control of steroidogenesis throughout pregnancy. This process of desensitization is a recognized phenomenon of many other G-protein coupled receptors (35) as well as kisspeptin (36).

ACTH secreted from the fetal pituitary is a crucial regulator of FA growth, partly mediated by peptide growth factors in an autocrine or paracrine fashion (13, 24). Placental CRH and estrogens may also play important roles in the development of the FA. However, the rapid growth and steroid output of the FZ during the second trimester are not paralleled by an increase in ACTH, and in humans CRH levels peak near parturition (13). This finding suggests that other pregnancy-specific factors regulate FZ growth and function, particularly in the second trimester.

It is established that ACTH and CRH directly promote DHEAS production from FA cells (15, 37), but the regulation of androgen production from FZ cells is not fully understood. One previous study has shown that kisspeptin can increase aldosterone production from H295R cells (24). Its role in the production of other steroidogenic hormones by the HFA has not previously been examined. Our ELISA data confirm that kisspeptin can significantly increase DHEAS production from H295R and 8- to 10-wpc and 15- to 20-wpc (10- to 22-weeks’ gestation) HFA cells. Analysis by LC-MS/MS showed a similar response for H295R. For 8- to 10-wpc HFA cells, the baseline values were much higher by ELISA, so that the calculated change was much higher. This difference is likely to be caused by interference in the ELISA from other steroid sulfates generated by this tissue. Paradoxically, DHEAS values after kisspeptin are lower by ELISA than by LC-MS/MS, which supports the concept that kisspeptin specifically stimulates DHEAS production. The kisspeptin effect was comparable to stimulation with ACTH or CRH alone. Furthermore, kisspeptin in combination with ACTH appears to augment this effect. In contrast, CRH in combination with kisspeptin significantly decreased DHEAS production. Thus kisspeptin may work in concert with CRH and ACTH to regulate HFA function and therefore the balance of estrogens during pregnancy. The placenta is the primary source of estrogen, and the concentration of estrogen increases with progressing GA. The timing of these interactions may be critical because ACTH levels remain fairly steady throughout pregnancy and circulating kisspeptin levels rise steadily between the first and third trimesters. Consequently, as pregnancy progresses, kisspeptin may work in tandem with ACTH to enhance DHEAS, the production of estrogens, and the maintenance of pregnancy. In support of this hypothesis, low levels of kisspeptin, particularly in early pregnancy, are associated with greater miscarriage risk (12).

CRH is postulated to play critical roles in fetal maturation and the onset of parturition. Consequently, the levels of CRH increase as pregnancy progresses and peak from 35 weeks’ gestation, corresponding with a fall in the level of cortisol-binding protein. Abnormal activation of the fetal hypothalamic–pituitary–adrenal axis and enlargement of the FAV have been associated with impending preterm birth (20, 21). It is feasible that kisspeptin modulates the effects of CRH in midgestation; however, its role in late pregnancy, when CRH is critical, warrants investigation.

Three-dimensional ultrasonography is an established and accurate method of assessing fetal organ volumes. More recently it has been reported as a reliable technique to measure HFA volume, with good intraobserver and interobserver repeatability (30, 38). One cross-sectional study reports correlations between FAV and efw and between FAV and GA (39). Interestingly, our data suggest that FAV was independent of both GA and efw. This discrepancy may be explained by the fact that our data were longitudinal and are therefore is more likely to show true correlations. Chang *et al.* (39) observed larger FAVs than those obtained in the current study, and two other groups report slightly lower FAVs at comparable GAs (20, 38). These differences may be attributed to different methods and inclusion criteria used. Additionally, all three studies report cross-sectional rather than longitudinal data. Importantly, the FAV measurements in the current study are in agreement with data obtained from a detailed postmortem study (40).

Circulating kisspeptin concentrations increase dramatically during pregnancy, and its levels reflect the amount of viable placental tissue (8). Consequently, a decline in the levels may be associated with increased miscarriage and preeclampsia risk (10, 12). This association was also demonstrated in twin pregnancies, where the death of one twin was associated with lower kisspeptin levels (12). Serial measurements of plasma kisspeptin in pregnant women have not previously been undertaken, but cross-sectional data suggest that the levels increase as pregnancy progresses (8). We report a significant increase in circulating kisspeptin in pregnant women between 20 and 28 weeks’ gestation, which correlates with the second-trimester rise in FAV. This increase in FAV is independent of sex and efw. It also coincides with the *in vitro* data, which show a significant increase in *Kiss1R* mRNA expression in second-trimester (13 to 22 weeks’ gestation) HFA cells and DHEAS production from midtrimester (10 to 22 weeks’ gestation) HFA cells after kisspeptin treatment.

## Conclusions

Kisspeptin–Kiss1R signaling may be a key regulator of HFA development and steroidogenesis and therefore an integral component of the fetoplacental unit. As well as being critical in regulating placentation in early pregnancy, it may have a key physiological role in intrauterine homeostasis and the maintenance of pregnancy, particularly in the second trimester. Therefore, our data suggest a functional role for kisspeptin *in utero*.
