# Supplementary material for: Kisspeptin Is a Novel Regulator of Human Fetal Adrenocortical Development and Function: A Finding With Important Implications for the Human Fetoplacental Unit
Source: J Clin Endocrinol Metab. 2017 Jun 21;102(9):3349–59. doi: 10.1210/jc.2017-00763 (PMC5587078; doi:10.1210/jc.2017-00763)
Supplement: Supplementary file 1 [file jc.2017-00763.st1.docx]

**Supplemental Table 1. Details of antibodies used for immunofluorescence studies**

| Ab | Antigen and  fluorophore | Species | Company  (product number) | Dilution |
| --- | --- | --- | --- | --- |
| 1° | SF1 | Mouse monoclonal | Invitrogen (434200) | 1 in 200 |
| 2° | AF488-Green | Goat anti-mouse | Invitrogen (A11029) | 1 in 1000 |
| 1° | Kiss1R | Rabbit polyclonal | Alomone (AKR-001) | 1 in 100 |
| 2° | CY3-Red | Donkey anti-rabbit | Jackson Immunoresearch (711-165-152) | 1 in 1000 |
| 1° | SULT2A1 | Rabbit polyclonal | Abcam (38416) | 1 in 200 |
| 2° | CY3-Red | Donkey anti-rabbit | Jackson Immunoresearch (711-165-152) | 1 in 1000 |
| 1° | CD56-conjugated AF488-Green | Mouse monoclonal | Invitrogen MHCD5620 | 1 in 1000 |

Ab, antibody; 1°, primary antibody; 2°, secondary antibody.

**Supplemental Methods**

*DHEAS measurements using liquid chromatography-tandem mass spectrometry (LC-MS/MS)*

500μl of precipitation reagent (a cocktail of internal standards in acetonitrile, including 16,16 d_2_ DHA sulfate) was added to the samples, calibrators and internal quality controls (500μl). The quality controls were created in-house from a standard solution of DHA sulfate diluted in charcoal-stripped serum. Samples were vortexed (30 seconds) and centrifuged (13 000rpm) for 5 minutes. The supernatant was transferred into glass tubes. Bicarbonate solution (200μl, 8% aq, v/v) and 1ml of ethyl acetate was added and the tubes were vortexed (30 seconds) and centrifuged (13 000 rpm) for 5 minutes. The organic layer was transferred into a glass tube and evaporated to dryness under nitrogen gas and reconstituted in 125μl of freshly prepared reconstitution solution (Mobile phase A: Mobile phase B, 65:35 (v/v)). 100μl was injected onto the liquid chromatography (LC) system. Using the TSQ Vantage (ThermoFisher) in MS/MS positive APCI mode, *m/z* transitions 271.1 to 105 & 91 were monitored. DHEA was also quantified in the same runs and remained near or below detection limits, indicating that there was no significant desulfation during sample processing. Mass spectrometry parameters are listed in Supplemental Table 2 and method validation data is listed in Supplemental Table 3. Comparison of ELISA and LC-MS/MS DHEAS concentrations are given in Supplemental Table 4.

*Liquid chromatography conditions*

Eluents: Mobile Phase A: Water with 0.1 % formic acid

Mobile Phase B: Methanol with 0.1 % formic acid

Flow rate: 0.4ml/min

Column: Accucore RP-MS Column (100 x 2.1mm. 2.6 µm)

Column temperature: 40 ºC (maintained by Hot Pocket. ThermoScientific

**Supplemental Table 2. Mass spectrometry parameters**

| Vaporizer temperature | 500^o^C |
| --- | --- |
| Capillary temperature | 400^o^C |
| Discharge current (mA) | 5.0 |
| Sheath gas | 20 |
| Aux gas | 5 |
| Collision gas pressure (mTorr) | 1.5 |
| Q1 (FWHM): | 0.40 |
| Q3 (FWHM): | 0.70 |
| Scan time | 0.05 seconds |

**Supplemental Table 3. DHEAS assay validation data**

**A. Precision and accuracy**

| **Concentration nmol/L (n=6)** | **102** | **204** | **1697** | **8148** |
| --- | --- | --- | --- | --- |
| **Intra-assay** | | | | |
| **Mean** | **99.8** | **187.3** | **1840** | **8080** |
| **Accuracy %** | **97.9** | **92** | **108** | **99** |
| **CV%** | **5.3** | **3.0** | **3.8** | **1.3** |
| **Inter-assay** | | | | |
| **Mean** | **102.3** | **201.7** | **1789.7** | **7700.8** |
| **Accuracy %** | **100.3** | **98.9** | **105.5** | **94.5** |
| **CV%** | **8.1** | **3.7** | **5.6** | **4.7** |

Lower limit of quantification is 35 nmol/L. No carry over or carry under was detected. No Ion suppression/enhancement of DHEAS-d2 signal was noted

**B. Reagent stability**

| **Concentration nmol/L (n=3)** | **204** | **1697** | **5729** |
| --- | --- | --- | --- |
| **Post extraction 1 week at 4°C** | | | |
| Mean | 201.4 | 1646 | 5720 |
| Accuracy % | 96.7 | 97.0 | 99.8 |
| **Post extraction 1 week at room temperature** | | | |
| Mean | 202.4 | 1570 | 5539.3 |
| Accuracy % | 99.2 | 92.5 | 96.7 |
| **3 freeze-thaw cycles** | | | |
| Mean | 203.2 | 1633 | 5581 |
| Accuracy % | 99.6 | 96.2 | 97.4 |

**Supplemental Table 4. ELISA vs LC-MS/MS data**

**A. H295R cells**

|  | **DHEAS concentration ng/ml** | **DHEAS concentration ng/ml** | **DHEAS concentration ng/ml** | **Mean DHEAS concentration ng/ml** | **Mean fold change*** |
| --- | --- | --- | --- | --- | --- |
| **Un (ELISA)** | 105.9 | 141.8 | 110.4 | 119.4 | **1.0** |
| **KP (ELISA)**  **Fold increase** | 393.16  3.7 | 551.5  3.8 | 392.9  3.5 | 445.9 | **3.7** |
| **Un (LCMS)** | 129.8 | 168.0 | 12.5 | 103.4 | **1.0** |
| **KP (LCMS)**  **Fold increase** | 603.6  4.6 | 775.3  4.6 | 196.9  15.7 | 525.3 | **8.3** |

**B. 8-10wpc HFA cells**

|  | **DHEAS concentration ng/ml**  **8wpc HFA** | **DHEAS concentration ng/ml**  **9wpc HFA** | **DHEAS concentration ng/ml**  **10wpc HFA** | **Mean DHEAS concentration ng/ml**  **8-10wpc HFA** | **Mean fold change*** |
| --- | --- | --- | --- | --- | --- |
| **Un (ELISA)** | 138.9 | 85.6 | 100 | 108.2 | **1.0** |
| **KP (ELISA)**  **Fold increase** | 255.8  1.8 | 270.1  3.2 | 301.2  3.0 | 275.7 | **2.7** |
| **Un (LCMS)** | 2.92 | 4.8 | 2.32 | 3.3 | **1.0** |
| **KP (LCMS)**  **Fold increase** | 86.5  29.6 | 552.8  115.2 | 312.5  134.7 | 317.2 | **93.2** |

Un, unstimulated cells; KP, cells treated with 100nM kisspeptin. * Fold change calculated relative to untreated samples which are normalized to 1.0.

**Supplemental Table 5.** Patient demographics, gestation, fetal adrenal volume and kisspeptin levels at each antenatal visit.

|  | | | | Visit 1 | | | Visit 2 | | | Visit 3 | | | Visit 4 | | |
| --- | --- | --- | --- | --- | --- | --- | --- | --- | --- | --- | --- | --- | --- | --- | --- |
| Pt | **Age** | **Ethnicity** | **Gravidity/**  **Parity** | **GA (wks)** | **FA vol (cm^3^)** | **KP**  **(pmol/)** | **GA (wks)** | **FA Vol (cm^3^)** | **KP**  **(pmol/)** | **GA (wks)** | **FA Vol (cm^3^)** | **KP**  **(pmol/)** | **GA (wks)** | **FA Vol (cm^3^)** | **KP**  **(pmol/)** |
| 1 | 24 | O | G1, P0 | 19.43 | 0.34 | 2938.2 | 26.43 | 1.45 | 3680.5 | - | - | 5159.5 | 37.71 | 3.27 | 4997.9 |
| 2 | 29 | WE | G1, P0 | - | - | 2525.1 | 27.00 | 0.16 | 2524.2 | 33.00 | 1.17 | 4284.0 | 38.00 | 8.64 | 2210.0 |
| 3 | 34 | FEA | G1, P0 | 20.14 | 1.23 | 5719.2 | 27.14 | 3.18 | 5957.4 | 33.57 | 4.84 | 6686.0 | 37.57 | 0.82 | 5515.9 |
| 4 | 28 | AC | G4, P1 | 20.71 | 0.82 | 2131.5 | 27.71 | 1.16 | 5301.0 | 36.00 | 2.41 | 5092.3 | 39.71 | 13.61 | 3968.5 |
| 5 | 29 | SEA | G2, P1 | 20.43 | 0.48 | 2981.7 | 27.29 | 4.94 | 3130.9 | 34.29 | 0.57 | 6164.7 | 38.29 | 1.38 | 3338.8 |
| 6 | 28 | WE | G3, P1 | 20.29 | 1.32 | 2383.4 | 28.86 | 0.21 | 3718.4 | 34.00 | 9.11 | 4924.3 | 37.86 | 7.95 | 3970.8 |
| 7 | 32 | WE | G1, P0 | 19.29 | 0.07 | 2367.2 | 29.29 | 1.21 | 5615.0 | 34.71 | 1.03 | 6235.0 | 38.71 | 7.87 | 3702.8 |
| 8 | 34 | WE | G1, P0 | 20.57 | 0.15 | 4355.9 | 28.43 | 1.32 | 6381.8 | 34.57 | 4.45 | 7430.5 | 38.00 | 4.17 | 7774.3 |
| 9 | 30 | AC | G3, P1 | 20.43 | 0.10 | 1679.5 | 28.29 | 0.24 | 3273.3 | - | - | 2992.9 | 38.29 | 1.67 | 2883.1 |
| 10 | 20 | AC | G1, P0 | 20.00 | 0.55 | 3470.0 | 27.71 | 2.83 | 6500.8 | 33.71 | 2.08 | 6805.1 | 37.14 | 2.16 | 5967.5 |
| 11 | 35 | WE | G1, P0 | 22.43 | 0.15 | 2979.1 | 28.29 | 2.76 | 6574.4 | 34.43 | 4.81 | 6890.9 | 37.71 | 9.60 | 4818.6 |
| 12 | 30 | SEA | G1, P1 | 21.00 | 0.12 | 3885.0 | 29.86 | 2.90 | 2252.6 | 35.00 | 1.92 | 5962.4 | 37.43 | 1.17 | 3619.7 |
| 13 | 25 | SEA | G2, P0 | 20.14 | 0.08 | 1974.6 | 28.00 | 0.24 | 1428.8 | 34.00 | 1.50 | 3235.8 | 38.00 | 8.60 | 2107.7 |
| 14 | 31 | SEA | G2, P1 | - | - | 3370.5 | 29.14 | 2.80 | 5278.9 | 36.14 | 9.50 | 915.9 | - | - | - |
| 15 | 37 | WE | G1, P0 | 20.14 | 0.08 | 2933.6 | 27.71 | 3.15 | 4217.3 | 33.71 | 2.37 | 4159.6 | 37.86 | 5.73 | 4916.2 |
| 16 | 29 | SEA | G3, P2 | 20.43 | 0.43 | 1850.8 | 28.57 | 1.13 | 3965.1 | 34.71 | 1.71 | 4805.9 | 38.86 | 2.16 | 1977.3 |
| 17 | 32 | FEA | G3, P1 | 20.14 | 0.23 | 1541.0 | 26.29 | 0.36 | 1976.4 | 34.43 | 2.29 | 2506.1 | 38.29 | 2.70 | 3718.1 |
| 18 | 31 | M | G1, P0 | 20.14 | 0.49 | 3437.8 |  |  |  |  |  |  |  |  |  |
| 19 | 31 | SEA | G1, P0 | 20.00 | 0.08 | 3254.3 | 28.00 | 0.71 | 3918.9 | 34.14 | 1.34 | 3998.8 | 38.00 | 1.65 | 2613.1 |
| 20 | 25 | M | G1. P0 | 20.00 | 0.88 | 3648.5 | 27.71 | 1.56 | 5721.3 | 34.71 | 4.36 | 6649.5 | 37.86 | 0.75 | 5566.2 |
| 21 | 31 | WE | G1, P0 | 20.29 | 0.35 | 2949.1 | 28.29 | 0.45 | 2823.1 |  |  |  |  |  |  |
| 22 | 28 | SEA | G4, P2 | 20.43 | 0.19 | 1624.2 | 28.43 | 0.44 | 1098.3 | 34.43 | 0.95 | 1396.8 |  |  |  |
| 23 | 29 | WE | G2, P0 | 22.57 | 0.41 | 1781.0 | 27.71 | 1.4 | 4484.9 | 33.71 | 1.71 |  |  |  |  |
| 24 | 32 | M | G2, P1 | 20.43 | 0.13 | 2822.2 | 28.43 | 0.42 | 3666.8 | 34.29 | 1.06 | 5903.8 | 37.29 | 1.44 | 4683.6 |
| 25 | 24 | WE | G1, P0 | 19.57 | 0.10 | 3405.2 | 28.71 | 0.29 | 6034.0 | 35.29 | 0.31 | 6696.1 | 38.29 | 0.78 | 5560.9 |
| 26 | 30 | BA | G3, P2 | 20.43 | 0.12 | 2017.6 | 28.43 | 0.32 | - | - | - | 1678.7 | 37.71 | 0.88 | 2942.8 |
| 27 | 25 | WE | G1, P0 | 20.14 | 0.20 | 1542.9 | 28.29 | 0.59 | 2081.8 | 34.86 | 0.44 | 4098.7 | 39.29 | 2.36 | 3779.0 |
| 28 | 17 | SEA | G1, P0 | 19.86 | 0.01 | 3207.7 | 27.57 | 0.10 | 3358.0 | 34.71 | 0.31 | 3460.7 | 38.71 | 0.33 | 1694.6 |
| 29 | 17 | FEA | G2, P1 | 19.86 | 0.04 | 3276.0 | 27.86 | 0.33 | 5476.7 | 35.71 | 0.94 | 5520.2 | - | - | - |
| 30 | 17 | SEA | G2, P0 | 20.71 | 1.17 | 1793.8 | 28.57 | 0.25 | 3952.7 | 33.43 | 0.80 | 2568.6 |  |  |  |
| 31 | 17 | BA | G2, P0 | 20.14 | 0.21 | 2507.7 | 27.86 | 0.06 | 4959.1 | 34.00 | 0.84 | 3812.6 | - | - | 2345.8 |
| 32 | 17 | FEA | G1, P0 | 20.57 | 0.02 | 2403.9 | 28.14 | 0.33 | 5687.2 | 34.43 | 1.55 | 4103.6 | - | - | 3218.0 |
| 33 | 17 | SEA | G2, P0 | 20.14 | 0.07 | 882.0 | 28.14 | 0.10 | 2169.7 | 33.71 | 1.04 | 3019.1 | - | - | 2051.3 |

Pt, patient number; -, data missing (subjects failed to attend for scans / blood test); we, white European; fea, Far East Asian; sea, south east Asian; bc, black Caribbean; ba, black African; m, mixed race; o, other; GA, gestation age. Subject numbers 21 and 23 moved area before completion of the study (after visits 2 and 3, respectively). Subjects 18 and 30 had premature delivery at 26 and 33 weeks gestation, respectively.

**Supplemental Figure 1 legend**

**A-C.** Kisspeptin dose response studies. DHEAS production **(**ELISA) by H295R and HFA cells following kisspeptin treatment. H295R cells (B), 8-10wpc HFA cells (C) and 15-20wpc HFA cells (D) were incubated with kisspepin 1nM, 10nM, 100nM, or no treatment (-) for 24 hours. Data points are mean +/- SD from 3 independent experiments run in triplicate and expressed as the fold over basal level (normalized to a value of 1). (-), no treatment; (+) treatment added. **D**. Immunofluorescence studies of 11 wpc HFA. Localization of SF1 positive steroidogenic cells (green) is demonstrated throughout the cortex (panels a-c). Localization of Kiss1R (red) is detected throughout the FZ, as well as the DZ/TZ (panels a-c). Panel a, low power (x2.5); boxed section, high power (x40). The adrenal cortex is surrounded by an outer mesenchymal capsule (dashed line). Scale bar: 100μm. **E-F**. DHEAS production (LC-MS/MS) by H295R (E) and 8-10wpc HFA cells (F) following 24 hours 100nM kisspeptin treatment. Data points are mean +/- SD derived from 3 independent experiments run in triplicate and expressed as the fold over basal level (normalized to a value of 1). *p<0.05; **p<0.01; ****p<0.0001.
