# Supplementary material for: Kisspeptin Is a Novel Regulator of Human Fetal Adrenocortical Development and Function: A Finding With Important Implications for the Human Fetoplacental Unit
Source: J Clin Endocrinol Metab. 2017 Jun 21;102(9):3349–59. doi: 10.1210/jc.2017-00763 (PMC5587078; doi:10.1210/jc.2017-00763)
Supplement: Supplementary file 2 [file jc.2017-00763.sf1.pptx]

## Slide 1
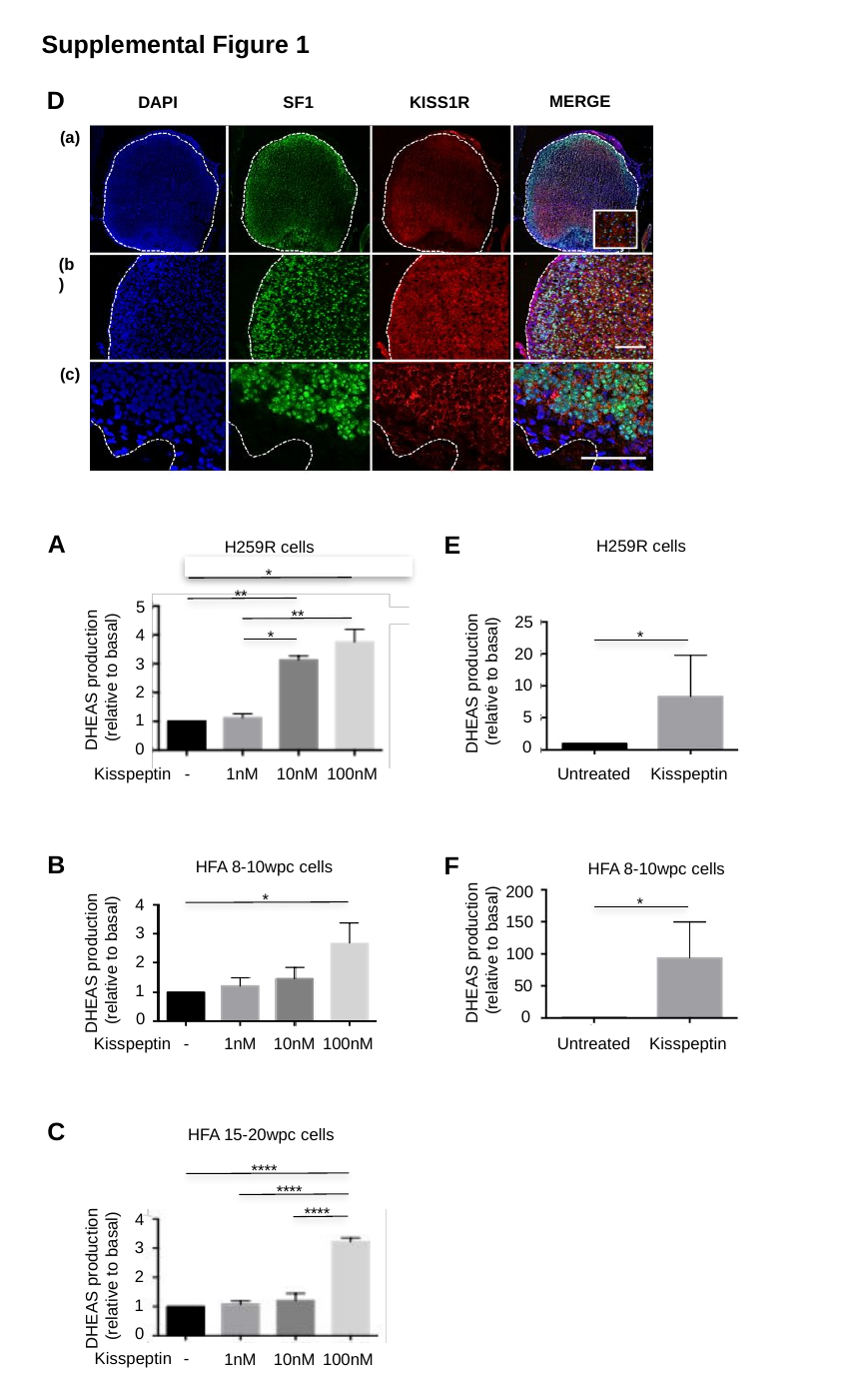

Supplemental Figure 1
D
MERGE
DAPI
SF1
KISS1R
(a)
(b)
(c)
A
E
H259R cells
H259R cells
*
**
5
**
25
4
*
*
20
3
DHEAS production
(relative to basal)
DHEAS production
(relative to basal)
10
2
5
1
0
0
-
10nM
Untreated
Kisspeptin
100nM
Kisspeptin
1nM
B
F
HFA 8-10wpc cells
HFA 8-10wpc cells
200
*
*
4
150
3
DHEAS production
(relative to basal)
DHEAS production
(relative to basal)
100
2
50
1
0
0
Kisspeptin
-
10nM
Untreated
100nM
Kisspeptin
1nM
C
HFA 15-20wpc cells
****
****
****
4
3
DHEAS production
(relative to basal)
2
1
0
-
Kisspeptin
10nM
100nM
1nM
